# Association Between Foramen Rotundum and Trigeminal Neuralgia in the Saudi Population: A Radiological Study

**DOI:** 10.7759/cureus.51932

**Published:** 2024-01-09

**Authors:** Essam E Ismail, Mohammed S Alaftan, Rinad M Aljoaid, Fatima M Al Musabeh, Sana M Alaidarous, Deem Hamad Alsultan, Mohammed A Alammari, Sanket D Hiware, V. Christopher Amalraj, Ujwal Gajbe, Brij Raj Singh

**Affiliations:** 1 Anatomy, College of Medicine, Imam Abdulrahman Bin Faisal University, Dammam, SAU; 2 Radiology, College of Medicine, Imam Abdulrahman Bin Faisal University, Dammam, SAU; 3 Anatomy, Graphic Era Institute of Medical Sciences, Dehradun, IND; 4 Development and Community, College of Medicine, Imam Abdulrahman Bin Faisal University, Dammam, SAU; 5 Anatomy, Datta Meghe Medical College, Datta Meghe Institute of Higher Education and Research, Nagpur, IND

**Keywords:** cranial anatomy, skull, morphological variations, foramen rotundum, trigeminal neuralgia

## Abstract

The trigeminal nerve is responsible for transmitting sensory information from the face, nasal and mouth cavities, and most of the scalp. Trigeminal neuralgia (TN) is a chronic facial pain disorder characterized by spontaneous paroxysmal pain throughout the distribution of the trigeminal nerve. This study investigated the morphological and morphometric variations of the foramen rotundum (FR) and its association with TN through a retrospective radiological analysis. A cohort of 97 participants from King Fahad University Hospital, Saudi Arabia, comprising 57 TN patients and 40 controls, underwent head CT scans for measurement and analysis. The study revealed significant differences in the FR morphology between TN patients and controls, particularly noting narrower FR measurements among TN individuals, especially in females. The right side demonstrated narrower FR dimensions, potentially correlating with the predominant side of pain in TN patients. While the presence of bony spurs was absent in all participants, variations in FR shape, size, and spatial positioning were observed and compared across genders and groups. These findings provide crucial insights into the potential anatomical factors contributing to TN, emphasizing the importance of understanding FR variations in clinical assessment and management of TN cases. Further research focusing on FR morphology and its clinical implications is recommended to enhance understanding and aid medical professionals in addressing TN-related concerns.

## Introduction

The largest cranial nerve, the trigeminal, is responsible for transmitting sensory data from the face, nasal and mouth cavities, and most of the scalp. The three divisions of the trigeminal nerve are the ophthalmic, maxillary, and mandibular nerves [1]. These divisions emerge from the cranial cavity through the supraorbital fissure, foramen rotundum (FR), and foramen ovale, respectively [2,3]. The FR is located in the greater wing of the sphenoid bone at the base of the skull, medially positioned to the foramen ovale [4,5]. The maxillary nerve passes through the FR, which can undergo compression due to the possible presence of the bony spur [6]. There are morphological and anatomical variations that can occur for developmental reasons. In addition, different bone structures exist, including the bony spine, plate, and tubercles, and variations in shape are present. These differences could be due to variations in the ossification of the greater wings of the sphenoid bone during the development process. Therefore, it is important for clinicians to assess this variation, especially in the case of trigeminal neuropathy [7]. Trigeminal neuralgia (TN) is a particular type of facial pain syndrome characterized by spontaneous paroxysmal pain throughout the distribution of the trigeminal nerve, mainly in the maxillary and mandibular divisions [8]. Trigeminal nerve compression caused by a blood vessel is the main cause of TN even in asymptomatic patients [9]. Anatomical and radiological studies have reported that maxillary nerve compression, as it passes through the FR, is another suspected cause of TN [10,11]. The current study is crucial to understanding the relationship between FR shape and size and TN, using high-resolution computed tomography (CT), in the Saudi population.

## Materials and methods

Study design

A retrospective study was carried out on head, mastoid, and sphenoid sinus CT images, in the Department of Radiology, King Fahad University Hospital (KFUH), Al Khobar, Eastern Province, Saudi Arabia. The study included individuals without head or neck pathologies or symptoms, as well as patients with TN. CT images were obtained randomly, and had been performed for medical or surgical reasons in Saudi patients. However, images containing motion artifacts or pathologies in the FR were excluded. Measurements were taken from 97 CT images by a radiologist, after obtaining approval from the Institutional Review Board at Imam Abdulrahman Bin Faisal University (no. IRB-UGS-2022-01-419).

The study included a cohort of 97 participants, consisting of 57 individuals diagnosed with TN and 40 without this condition. The data was sourced from the KFUH radiological database. A comprehensive examination of the right and left foraminal regions of the FR was carried out.

The inclusion criteria encompassed individuals meeting two specific parameters: having undergone a head CT examination at KFHU and falling within the age range of 22 to 50 years. The exclusion criteria encompassed individuals falling below 22 years or exceeding 50 years of age, those afflicted with neurological pathologies, or having undergone significant neurosurgical procedures.

Examination of FR dimensions

Examination and analysis of the dimensions and configurations of the FR derived from stored CT scans was done. The height, width, and distance from the midline of the right and left FRs were measured on coronal images. The dimensions of the FR were acquired through the utilization of a multidetector row CT scan, specifically employing the Somatom Definition apparatus (Siemens Healthcare, Forchheim, Germany). CT imaging specifications were configured with a section thickness of 0.6 mm, an energy level of 120 KVp, and a current range of 150-180 mA. The GE picture archiving and communication system (PACS) software (GE HealthCare, Chicago, IL) facilitated the measurement procedures, in which axial and coronal images were utilized to manually ascertain the dimensions attributed to the FR.

**Figure 1:**
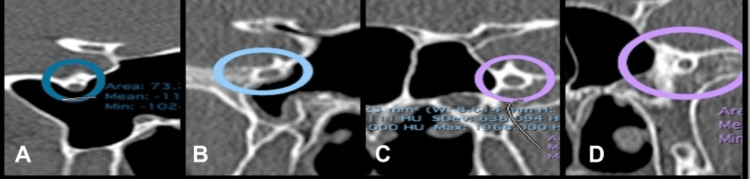
Types and positions of the foramen rotundum: (A) type I, (B) type IIa, (C) type IIb and (D) type III

The shape or location of the FR was classified as type I, IIa, IIb, or III according to its relationship with the sphenoid sinus. In type I, FR is located entirely within the cavity of the sphenoid sinus. In type IIa, a part of FR is partially protruding into the sinus; in type IIb, FR is parallel to the sinus wall; and in type III, FR is embedded within the sphenoid bone (Figure 1) [12]. Additionally, the presence of bony spurs was noticed.

Data analysis

The present investigation employed various statistical parameters in its data analysis. These included the mean (M), standard deviation (SD), standard error of the mean (SEM), and the 95% confidence interval (CI). An unpaired t-test was used to assess discrepancies in foramen measurements between both sides and between genders. Statistical significance was determined for all analyses in this study and was ascertained at a threshold P-value below 0.05. Furthermore, descriptive statistics were performed using IBM SPSS Statistics, version 22 for Windows (IBM Corp., Armonk, NY).

## Results

In this study, the demographic characteristics showed that the average age of males in the TN group was 35.33 years, compared to 34.08 years for females (Table 1). However, the mean age of males in the control group was 35.42 years versus 35.31 years for females. In terms of gender, the number of males was 21 (36.8%), compared to 36 (63.2%) females in the TN group. In the control group, there were 21 (52.5%) males and 19 (47.5%) females. Regarding the side of pain, most of the TN group had it on the right side (29, 50.9%) compared to patients with pain on the left side (22, 38.6%) and bilateral pain (6, 10.5%).

**Table 1:** Demographic analysis: gender and age distribution in TN and control groups n, number; TN, trigeminal neuralgia; SD, standard deviation

Groups and gender distribution	n	Minimum	Maximum	Mean	SD
TN group	57	22	50	34.08	10.46
Male	21	24	50	35.33	9.16
Female	36	22	50	34.08	10.46
Control group (without TN)	40	23	50	38.25	3.50
Male	21	24	48	35.42	2.82
Female	19	23	46	35.31	3.5

Regarding the shape of the FR in the TN group, it was observed that among females, the most frequent type for both sides was type IIb (65.2% for the left side and 50% for the right side). On the other hand, type III was the common type in males of the same group. Furthermore, type IIb was more frequent among males for the right side (76.2%) and females for the left side (68.4%) in the control group. Table 2 shows the FR shape variation between males and females for both sides.

**Table 2:** Foramen rotundum shape variation between males and females for both sides n, number; TN, trigeminal neuralgia

Groups	Gender	Type (left side)					Type (right side)				
		l, n (%)	lla, n (%)	llb, n (%)	lll, n (%)	Total, n (%)	l, n (%)	lla, n (%)	llb, n (%)	lll, n (%)	Total, n (%)
TN group (n = 57)	Male	-	3 (14.3)	8 (38.1)	10 (47.6)	21 (47.6)	1 (4.8)	3 (14.3)	5 (23.8)	12 (57.1)	21 (36.8)
	Female	2 (5.6)	8 (22.2)	15 (65.2)	11 (41.7)	36 (63.2)	-	5 (13.9)	18 (50)	13 (36.1)	36 (63.2)
Control group (n =40)	Male	-	1 (4.8)	13 (61.9)	7 (33.3)	21 (52.5)	-	1 (4.8)	16 (76.2)	4 (19)	21 (52.5)
	Female	-	1 (5.3)	13 (68.4)	5 (26.3)	19 (47.5)	-	1 (5.3)	11 (57.9)	7 (36.8)	19 (47.5)

A comparison between both sides in the TN group revealed that the width of the FR was insignificantly narrower on the right side in patients with TN. The length and distance from the midline was observed to be significantly higher on the right side in the TN group. In the control group, there were no significant differences in any measurements between the right and left sides. Table 3 shows a comparison of the measurements of the length, width, and distance from the midline for the foramen rotundum on both sides.

**Table 3:** A comparison of the length, width and distance from the midline for the foramen rotundum on both sides n, number; TN, trigeminal neuralgia; ns, not significant; SD, standard deviation; SEM, standard error of the mean; CI, confidence interval P-value <0.05 *Significant **Highly significant

Groups			Mean	n	SD	SEM	95% CI		t-test	P-value
							Lower	Upper		
TN group	Height	Right	0.691	57	0.293	0.039	-0.528	-0.303	7.416	0.000^**^
		Left	0.275	57	0.296	0.039				
	Width	Right	0.250	57	0.370	0.049	-0.228	0.024	1.629	0.109^ns^
		Left	0.256	57	0.592	0.078				
	Distance from the midline	Right	1.775	57	0.244	0.032	-0.122	-0.012	2.434	0.018^*^
		Left	1.708	57	0.277	0.037				
Control group	Height	Right	0.275	40	0.054	0.009	-0.006	0.004	-0.365	0.717^ns^
		Left	0.274	40	0.054	0.008				
	Width	Right	0.278	40	0.041	0.006	-0.007	0.019	0.940	0.353^ns^
		Left	0.381	40	0.048	0.008				
	Distance from the midline	Right	1.720	40	0.238	0.038	-0.022	0.072	1.068	0.292^ns^
		Left	1.695	40	0.249	0.039				

When comparing FR measurements between men and women in the TN group, it was found that the FR was significantly narrow among females, whereas the height and distance of FR from the midline were insignificantly lower in females of the TN group. In the FR control group, measurements did not show significant differences between males and females. Table 4 shows the measurement comparison between males and females for the foramen rotundum.

**Table 4:** Foramen rotundum measurement comparison between males and females n, number; TN, trigeminal neuralgia; ns, not significant; SD, standard deviation; SEM, standard error of the mean; CI, confidence interval P-value <0.05 *Significant

Unpaired sample statistics										
Groups			n	Mean	SD	SEM	95% CI		t-test	P-value
							Lower	Upper		
TN group	Height	Male	21	0.281	0.022	0.005	-0.123	0.150	1.360	0.179^ns^
		Female	36	0.268	0.368	0.061				
	Width	Male	21	0.257	0.042	0.080	0.008	0.062	3.672	0.000^*^
		Female	36	0.214	0.043	0.070				
	Distance from the midline	Male	21	1.786	0.031	0.007	0.008	0.062	1.400	0.167^ns^
		Female	36	1.646	0.032	0.005				
Control group	Height	Male	21	0.220	0.065	0.014	-0.022	0.047	0.747	0.460^ns^
		Female	19	0.385	0.039	0.009				
	Width	Male	21	0.259	0.047	0.010	-0.029	0.022	0.258	0.798^ns^
		Female	19	0.254	0.030	0.007				
	Distance from the midline	Male	21	1.718	0.250	0.054	-0.128	0.173	0.300	0.765^ns^
		Female	19	1.696	0.217	0.050				

When comparing the FR measurements in both groups, the height, width and distance from the midline in males in the TN group were insignificantly higher than those of the control group. However, the width of FR in the females of the TN group was not significantly narrower than the females of the control group. Similarly, the FR was significantly higher and more distant from the midline in the females in the control group (Table 5). In this study, all participants in both groups showed no bony spur in the FR (Table 6).

**Table 5:** Comparison of FR measurements between groups TN, trigeminal neuralgia; ns, not significant; FR, foramen rotundum P-value <0.05

Item	TN group		Control group	
	Male	Female	Male	Female
Height	0.281 (0.508)	0.268 (0.046)	0.220 (0.065)	0.385 (0.039)
	P = 0.357^ns^			
Width	0.257 (0.042)	0.214 (0.043)	0.259 (0.47)	0.254 (0.030)
	P = 0.953^ns^			
Distance from the midline	1.786 (0.190)	1.646 (0.260)	1.718 (0.250)	1.696 (0.217)
	P = 0.327^ns^			

**Table 6:** Presence of spur in the foramen rotundum TN, trigeminal neuralgia

	Side	Categories	Frequency	%
TN group	Right	Absent	57	100.0
	Left	Absent	57	100.0
Control group	Right	Absent	40	100.0
	Left	Absent	40	100.0

## Discussion

In this retrospective study, data was obtained using CT scans of 97 patients that measured the morphological aspects of the FR. Out of the 97, 57 were diagnosed with TN and 40 comprised the control group. In consistency with this research, Aksoy et al. evaluated the length, width, distance from the midline for foramen ovale (FO) and FR in patients with trigeminal neuralgia using CT and cone-beam CT [8]. In contrast, Elnashar et al. [4], Srimani et al. [9], Akcay et al. [10] and Reymond et al. [11] used dried skulls for the same measurements; this may cause differences in the findings.

The current study reported that most of the patients with TN had pain on the right side. This finding may be supported by the hypothesis of Neto et al. who stated that the FR of the human skull is significantly narrower on the right side, and FR provides passage for the maxillary nerve, which is commonly affected in TN [13].

Regarding the shape of the FR, this work indicated that the most common shape in the TN group was type III in males and type IIb in females on both sides. However, the most common shape in the control group was IIb on both sides and in both genders. Edouard et al. stated that the most common shape of the FR was IIb in males and III in females for both sides [12]. It is important to note that differences in the sample size and research region can alter the results.

The present research revealed that the width of the FR in patients with TN was narrower on the right side. This observation coincides with that of Liu et al. and Neto et al. who indicated that the right side is narrower than the left side in patients with TN [7,13]. In contrast, Mohebbi et al. found that the average right and left FR distances to the midline were 19.00 and 19.34 mm, respectively, which were significantly higher than the current results [14].

When correlating FR measurements, this study found that the length, width and distance from the midline were lower in females than in males in the TN group, except for the height of FR, which was lower in males. Specifically, this study reported a statistically significant narrowing of FR in females in the same group, which could explain the higher incidence of TN among females. These findings coincide with those of Liu et al. who stated that the narrow skull foramen may be a causal factor in TN patients [7]. When comparing FR measurements between the TN and control groups, the current research found that FR was insignificantly narrower in the TN group in both sexes compared to the control group. These results are consistent with the findings of Kastamoni et al. who concluded that the statistically significant smaller dimensions of FR and distances to the midline in patients with TN suggested that they might be an etiological factor for TN [15].

This study has certain limitations that need to be considered. First, the sample size is small due to the rarity of TN. Second, the research relied on a limited number of CT scans of patients diagnosed with TN that doesn’t usually require a CT scan for diagnosis. Last, the study was conducted at a single center, and therefore, the results may not be applicable to the entire population of patients with TN in Saudi Arabia.

## Conclusions

This retrospective study found that FR measurements were lower in individuals with TN compared to the control group, and lower in females than males in the TN group. Furthermore, the study identified that the right side was narrower than the left side, which may be related to the side of pain in TN. Further studies are recommended to focus on this topic to increase knowledge and assist medical professionals in the treatment of patients with TN.
